# Short Sleep Duration Is Associated with Reduced Leptin, Elevated Ghrelin, and Increased Body Mass Index

**DOI:** 10.1371/journal.pmed.0010062

**Published:** 2004-12-07

**Authors:** Shahrad Taheri, Ling Lin, Diane Austin, Terry Young, Emmanuel Mignot

**Affiliations:** 1Howard Hughes Medical Institute, Stanford UniversityPalo Alto, CaliforniaUnited States of America; **2**Department of Population Health Sciences, University of WisconsinMadison, WisconsinUnited States of America; Centre National de la Recherche Scientifique Institut de Biologie de LilleFrance

## Abstract

**Background:**

Sleep duration may be an important regulator of body weight and metabolism. An association between short habitual sleep time and increased body mass index (BMI) has been reported in large population samples. The potential role of metabolic hormones in this association is unknown.

**Methods and Findings:**

Study participants were 1,024 volunteers from the Wisconsin Sleep Cohort Study, a population-based longitudinal study of sleep disorders. Participants underwent nocturnal polysomnography and reported on their sleep habits through questionnaires and sleep diaries. Following polysomnography, morning, fasted blood samples were evaluated for serum leptin and ghrelin (two key opposing hormones in appetite regulation), adiponectin, insulin, glucose, and lipid profile. Relationships among these measures, BMI, and sleep duration (habitual and immediately prior to blood sampling) were examined using multiple variable regressions with control for confounding factors.

A U-shaped curvilinear association between sleep duration and BMI was observed. In persons sleeping less than 8 h (74.4% of the sample), increased BMI was proportional to decreased sleep. Short sleep was associated with low leptin (*p* for slope = 0.01), with a predicted 15.5% lower leptin for habitual sleep of 5 h versus 8 h, and high ghrelin (*p* for slope = 0.008), with a predicted 14.9% higher ghrelin for nocturnal (polysomnographic) sleep of 5 h versus 8 h, independent of BMI.

**Conclusion:**

Participants with short sleep had reduced leptin and elevated ghrelin. These differences in leptin and ghrelin are likely to increase appetite, possibly explaining the increased BMI observed with short sleep duration. In Western societies, where chronic sleep restriction is common and food is widely available, changes in appetite regulatory hormones with sleep curtailment may contribute to obesity.

## Abstract 2

The less people sleep the higher their risk for being overweight. This study shows that sleep duration affects appetite-regulating hormones

## Introduction

In population studies, a dose-response relationship between short sleep duration and high body mass index (BMI) has been reported across all age groups [1–7]. In the largest studied sample, elevated BMI occurred for habitual sleep amounts below 7–8 h [2]. A U-shaped curvilinear relationship between sleep duration and BMI was observed for women, but for men, there was a monotonic trend towards higher BMI with shorter sleep duration. Importantly, a recent prospective study identified a longitudinal association between sleep curtailment and future weight gain [6]. How sleep curtailment may interact with body weight is unknown, but hormones regulating appetite and energy expenditure may be involved.

A number of hormones may mediate the interactions between short sleep duration, metabolism, and high BMI. We hypothesized that the two key opposing hormones in appetite regulation, leptin and ghrelin [8,9], play a significant role in the interaction between short sleep duration and high BMI. Leptin is an adipocyte-derived hormone that suppresses appetite [10]. Ghrelin is predominantly a stomach-derived peptide that stimulates appetite [9,11]. Other mediators of metabolism that may contribute include adiponectin and insulin. Adiponectin is a novel hormone secreted by adipocytes and is associated with insulin sensitivity [12,13]. We investigated the associations among sleep duration (acute and habitual), metabolic hormones, and BMI in the population-based Wisconsin Sleep Cohort Study [14].

## Methods

### Overview

The institutional review board of the University of Wisconsin Medical School approved all protocols for the study, and informed consent was obtained from all participants. The Wisconsin Sleep Cohort Study is an ongoing longitudinal study of sleep habits and disorders in the general population [14]. Briefly, to construct a defined sampling frame, all employees aged 30–60 y of four state agencies in south central Wisconsin were mailed a survey on sleep habits, health, and demographics in 1989. Mailed surveys were repeated at 5-y intervals. A stratified random sample of respondents was then recruited for an extensive overnight protocol including polysomnography at baseline. Stratification was based on risk for sleep-disordered breathing (SDB), with an oversampling of habitual snorers to ensure an adequate distribution of SDB. Analyses were adjusted for the weighted sampling when appropriate. Recruitment for baseline studies was staggered to conduct seven studies per week; study entry and follow-up time thus varied within the cohort. Exclusion criteria included pregnancy, unstable cardiopulmonary disease, airway cancers, and recent upper respiratory tract surgery. The baseline response rate was 51%, with most refusals due to the inconvenience of sleeping away from home. Follow-up studies have been conducted at 4-y intervals, with up to three follow-up studies to date. Collection of morning, fasted blood was added to the protocol in 1995. Extensive survey and other data available from the sampling frame have been used to evaluate the potential for response and drop out biases.

**Figure 1:**
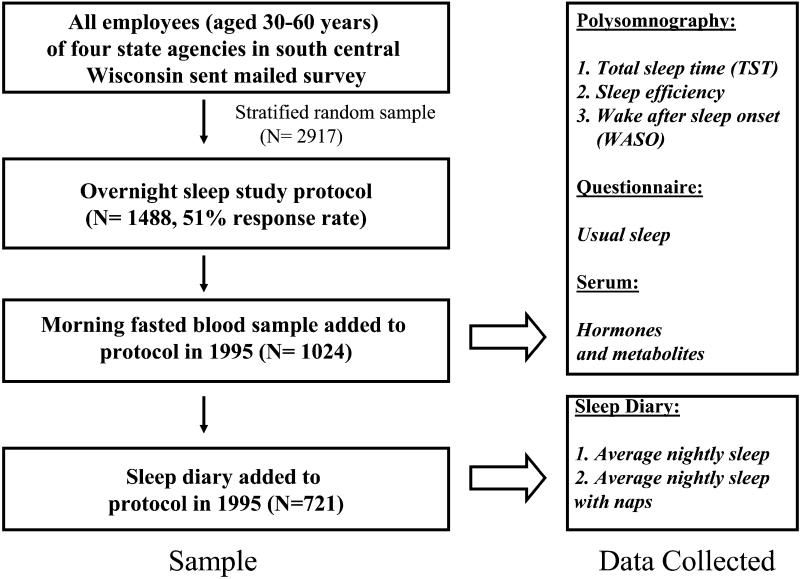
Sample Construction and Data Collected All employees (aged 30–60 y) of four state agencies in south central Wisconsin were mailed surveys starting in 1989 regarding general health and sleep habits. From this population, a stratified random sample of respondents was recruited for an extensive overnight protocol providing polysomnography and sleep questionnaire data and morning, fasted serum for hormone and metabolite measurement. The metabolic hormones measured were ghrelin (856 participants), leptin (1,017 participants), adiponectin (1,015 participants), and insulin (1,014 participants) (see Table 1). Based on scheduling availability, 721 participants completed an added protocol to measure daytime sleepiness that included a 6-d sleep diary, of which 714 reported on naps (see Table 1). See text for further description of the study population and definitions of the sleep measures used.

Figure 1 provides an overview of the study population and the data collected. The sample comprised 1,024 participants with an overnight study and blood sample. A 6-d diary of sleep duration was added as part of a protocol to assess daytime sleepiness after the initiation of the cohort study; these data were available for 721 participants.

**Table 1:**
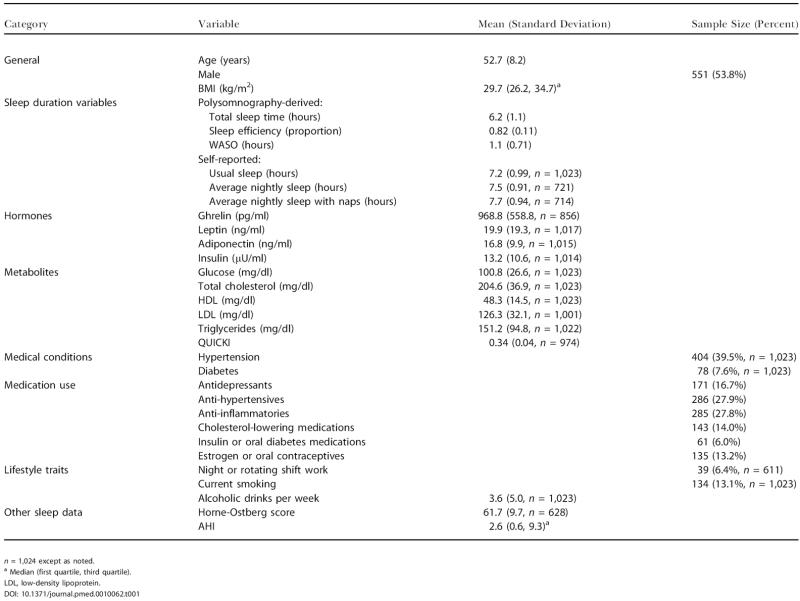
Characteristics of the Sample

*n =* 1,024 except as noted

^a^ Median (first quartile, third quartile)

LDL, low-density lipoprotein

DOI: 10.1371/journal.pmed.0010062.t001

### Data Collection

This investigation is based on Wisconsin Sleep Cohort Study data collected from the mailed sleep surveys, overnight studies, and 6-d sleep diaries. Overnight studies were conducted in laboratory bedrooms, with participants setting their own sleep and rise times. After informed consent was obtained, questionnaires on lifestyle and health history were administered, and height and weight measured. A blood sample was collected shortly after awakening from overnight polysomnography.

#### Polysomnography

An 18-channel polysomnographic system was used to assess sleep states, and respiratory and cardiac variables [14]. Sleep was studied using electroencephalography, electro-oculography, and chin electromyography (Grass Instruments, Quincy, Massachusetts, United States). Continuous measurement of arterial oxyhemoglobin saturation by pulse oximetry (Ohmeda, Englewood, Colorado, United States), oral and nasal airflow, nasal air pressure, and thoracic cage and abdominal respiratory motion (Respitrace Ambulatory Monitoring, Ardsley, New York, United States) were used to assess SDB [14]. Each 30-s interval of the polysomnographic record was scored for sleep stage and SDB using standard criteria [14]. The average number of apneas and hypopneas per hour of measured sleep, the apnea-hypopnea index (AHI), was the measure for SDB. For analyses including AHI as a covariate, participants were excluded if they had sleep studies with less than 4 h of usable polysomnography, or if they were receiving treatment for SDB.

#### Polysomnographic measures of acute sleep

Polysomnographic measures of sleep duration just prior to blood sampling were used to evaluate degree of “acute sleep restriction.” “Total sleep time” was total hours of polysomnographically defined sleep. “Wake after sleep onset” (WASO) was hours of wake time after three epochs of sleep had occurred. “Sleep efficiency” was total sleep time divided by time from lights out until arising in the morning.

#### Self-reported sleep measures of chronic sleep

Two variables were used to evaluate degree of “chronic sleep restriction” by estimating average nightly sleep duration: (i) “usual sleep” (from questionnaires) and (ii) “average nightly sleep” (from sleep diaries).

#### Questionnaire

Usual sleep was estimated from the following questions: how many hours of sleep do you usually get in (a) a workday night? (b) a weekend or non-work night? These questions were included in all mailed surveys and were added to questionnaires completed at the overnight study in 1998. For participants studied after 1998, data from questionnaires administered at the overnight study were used (58%); for the remainder of the sample, data from the mailed survey closest in time to the overnight study were used. Usual sleep was calculated as (5 × workday sleep + 2 × weekend sleep)/7.

#### Sleep diary

Average sleep duration was also estimated using a 6-d sleep diary, kept by 721 participants as part of an added protocol to measure daytime sleepiness. The median time between the blood collection and completion of the diary was 18 d. Almost all diaries (97%) were completed within 6 mo of blood sampling. In diaries, participants recorded the time they went to bed and arose each day, and the duration of any naps. “Average nightly sleep” was calculated as the sum of the hours between bedtime and arising divided by six. “Average nightly sleep plus naps” added naps to the above sum.

### The Relationship between BMI and Sleep Duration

For the analysis of the association of BMI and sleep duration, the sample comprised 1,040 participants with at least one 6-d sleep diary. Of these, 4-, 8-, and 12-y follow-up studies had been completed by 397, 179, and 11 participants respectively (1,828 visits), providing repeated measures data for greater analytic efficiency and precision.

### Hormone Assays

Following overnight fasting, serum was collected soon after awakening and stored at −70 °C. All samples were assayed in duplicate. It was not possible to assay samples from all participants in all assays because of the volume of serum available; this particularly affected the ghrelin assay, which required the most volume. Leptin and insulin were determined using enzyme-linked immunoassays (ELISA; Linco Research, St. Charles, Missouri, United States). Total ghrelin and adiponectin were measured by radioimmunoassay (Linco Research). Sensitivity for the leptin and insulin enzyme-linked immunoassays was 0.5 ng/ml and 2 μU/ml, respectively. Sensitivity for the ghrelin and adiponectin radioimmunoassays was 10 pg/ml and 1 ng/ml, respectively. Intra- and inter-assay variations were all less than 5% and less than 10%, respectively. The quantitative insulin sensitivity check index (QUICKI) was 1/(log (*I*) + log (*G*)), where *I* is fasting insulin and *G* is fasting glucose [15,16].

### Statistical Analysis

All analyses were cross-sectional and performed using SAS/STAT 8.2. Leptin, ghrelin, and adiponectin were square-root transformed and insulin log transformed based on the distribution of residuals from the multivariate regression models. We evaluated the relationship of age, sex, BMI, and time of storage of blood sample on hormones using multiple regression. Partial correlations adjusted for age, sex, and BMI were calculated for hormones and QUICKI, with and without control of other potential confounders. The relationships between hormones and sleep were evaluated using multiple linear regression after control for potential confounders including age, sex, BMI, SDB, and morningness tendencies (as measured using the Horne-Ostberg questionnaire, an indirect surrogate of earlier rising time). In all analyses involving insulin, glucose, and QUICKI (but not leptin, ghrelin, and adiponectin), participants with diabetes (self-reported diagnosis, or currently taking insulin or diabetic medications, or with glucose >300 mg/dl) were excluded. Participants with SDB were not removed from the analyses shown. When controlling for AHI in models, participants who used continuous positive airway pressure or who had inadequate sleep were excluded. Because controlling for AHI did not significantly change the sleep-hormone regression coefficients, these analyses are not shown. The relationship of BMI with average nightly sleep was evaluated using a quadratic fit. This was examined using multiple visits (*n =* 1,828) from 1,040 participants with sleep diary data available. Mixed modeling techniques were used to account for within-subject correlation for participants with multiple visits. SAS procedure mixed was used for modeling and hypothesis testing using robust standard errors and a compound symmetric within-subject correlation structure. All reported *p* values are two-sided. For illustrative purposes, changes in leptin, ghrelin, and BMI for different sleep amounts were calculated at the average values and sex distribution of the relevant sample.

## Results

**Figure 2:**
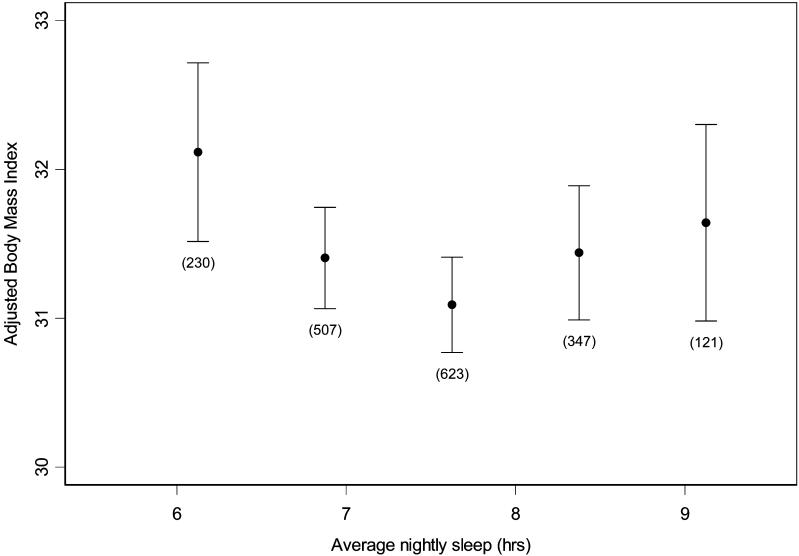
The Relationship between BMI and Average Nightly Sleep Mean BMI and standard errors for 45-min intervals of average nightly sleep after adjustment for age and sex. Average nightly sleep values predicting lowest mean BMI are represented by the central group. Average nightly sleep values outside the lowest and highest intervals are included in those categories. Number of visits is indicated below the standard error bars. Standard errors are adjusted for within-subject correlation.

Table 1 shows the characteristics of the sample, unadjusted for the weighted sampling scheme. Figure 2 shows the mean BMI for 45-min intervals of average nightly sleep after adjustment for age and sex. We found a significant U-shaped curvilinear relationship between average nightly sleep and BMI after adjustment for age and sex (average nightly sleep coefficient = −2.40, *p =* 0.008; (average nightly sleep)^2^ coefficient = 0.156, *p =* 0.008; the two coefficients define a curve). The minimum BMI was predicted at 7.7 h of average nightly sleep. The most striking portion of the curve was for persons sleeping less than 8 h (74.4% of the sample), where increased BMI was proportional to decreased sleep. An increase in BMI from 31.3 to 32.4 (+3.6%) corresponded approximately to an average nightly sleep duration decrease from 8 h to 5 h, as estimated at the mean age (53.1 y) and sex distribution (54.4% male) of the sample with available sleep diary data.

Table 2 shows the association of each of the hormones, glucose, and QUICKI with age, sex, and BMI. Serum ghrelin, leptin, adiponectin, insulin, and glucose were significantly correlated with BMI and sex. Storage time had significant effects on some but not all variables; in all cases, however, effect size was small, and the effect was corrected for in all calculations. Adiponectin and glucose were correlated with age. Table 3 shows partial correlations among the measured and calculated variables, adjusted for age, sex, and BMI. All correlations agree with previous studies and validate our assays and population sample. We also examined several potential confounders to be later controlled for, if needed, in our models. Identified relationships included: ghrelin with high-density lipoprotein (HDL), alcohol intake, and creatinine; leptin with diastolic blood pressure, smoking, and blood urea nitrogen (BUN); adiponectin with HDL, triglycerides, uric acid, and BUN; insulin with HDL, triglycerides, uric acid, and smoking; glucose with HDL, triglycerides, uric acid, alcohol intake, BUN, and creatinine; QUICKI with HDL, triglycerides, uric acid, smoking, and BUN. When diabetics (diagnosed) and participants with high glucose (glucose >300 mg/dl) were removed from this analysis, all relationships remained significant except for the correlation between ghrelin and adiponectin, and ghrelin and insulin.

**Table 2:**
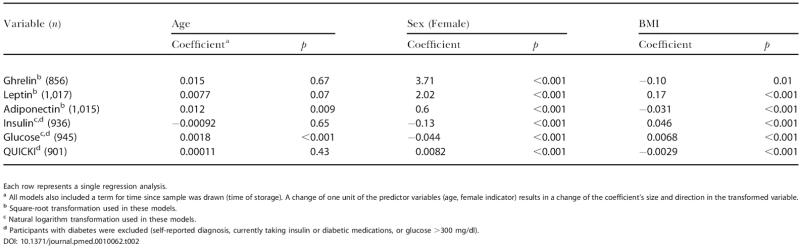
Relationships among Metabolic Hormone Levels, Age, Sex and BMI

Each row represents a single regression analysis

^a^ All models also included a term for time since sample was drawn (time of storage). A change of one unit of the predictor variables (age, female indicator) results in a change of the coefficient's size and direction in the transformed variable

^b^ Square-root transformation used in these models

^c^ Natural logarithm transformation used in these models

^d^ Participants with diabetes were excluded (self-reported diagnosis, currently taking insulin or diabetic medications, or glucose >300 mg/dl)

DOI: 10.1371/journal.pmed.0010062.t002

**Table 3:**
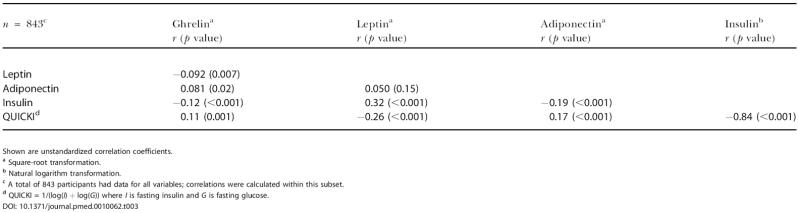
Partial Pearson Correlations of Metabolic Hormones and QUICKI after Adjustment for Sex, Age, and BMI

Shown are unstandardized correlation coefficients

^a^ Square-root transformation

^b^ Natural logarithm transformation

^c^ A total of 843 participants had data for all variables; correlations were calculated within this subset

^d^ QUICKI = 1/(log(*I*) + log(*G*)) where *I* is fasting insulin and *G* is fasting glucose

DOI: 10.1371/journal.pmed.0010062.t003

**Figure 3:**
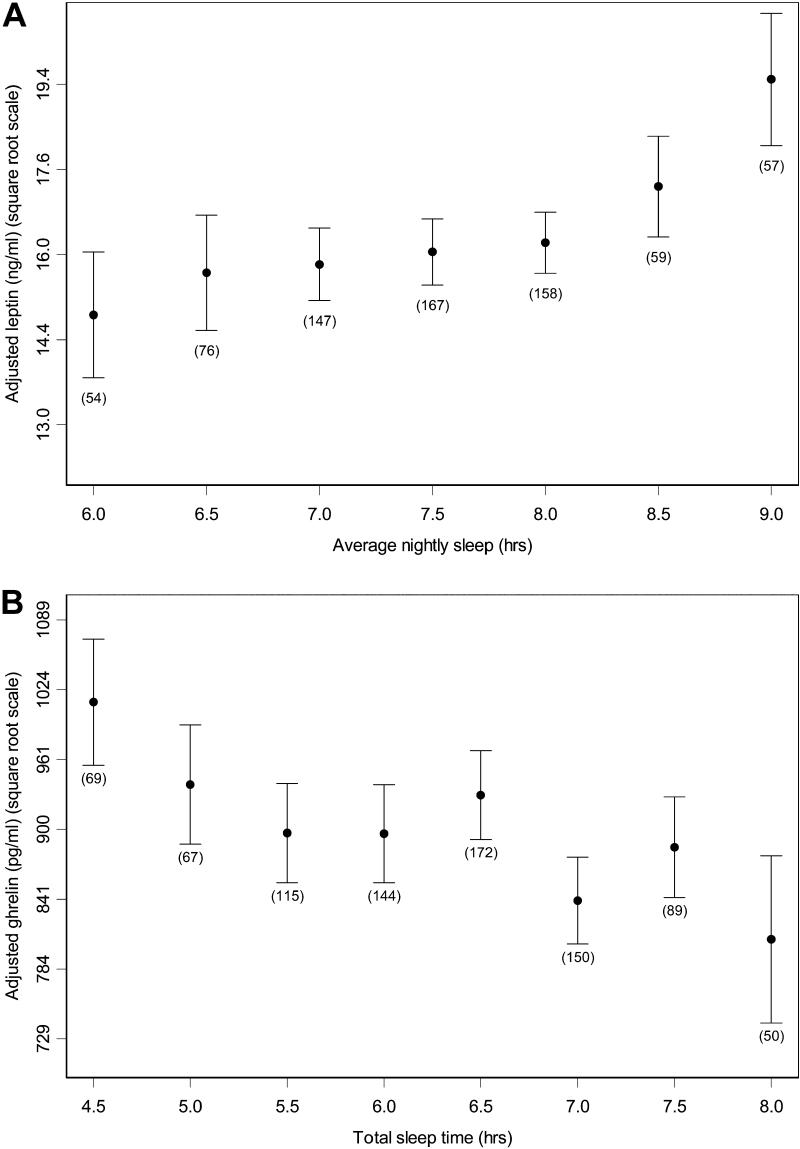
The Association between Sleep Duration and Serum Leptin and Ghrelin Levels (A) Mean leptin levels and standard errors for half-hour increments of average nightly sleep after adjustment for age, sex, BMI, and time of storage (see Table 2). Average nightly sleep values outside the lowest and highest intervals are included in those categories. Sample sizes are given below the standard error bars. The y-axis uses a square-root scale. Data derived from 718 diaries because three participants had missing leptin data. (B) Mean ghrelin levels and standard errors for half-hour increments of total sleep time after adjustment for age, sex, BMI, and time of storage (see Table 2). Total sleep time values outside the lowest and highest intervals are included in those categories. The y-axis uses a square-root scale. Note that ranges for total sleep time amounts are typically shorter than those for average nightly sleep amounts (A; see Figure 1), and do not correlate strongly (see text).

Using polysomnography to measure objective sleep immediately prior to blood sampling, ghrelin correlated significantly with total sleep time, sleep efficiency, and WASO (Table 4). Using questionnaire and diary data estimating chronic sleep, significant correlations were found between leptin and average nightly sleep (with and without naps) and usual sleep amounts (Table 4). A significant correlation was also observed between ghrelin and average nightly sleep plus naps. These relationships were consistently found when other possible confounding factors such as medications, hypertension, AHI, and factors listed above were included in the statistical model (analysis not shown). In unadjusted models, leptin was significantly correlated with total sleep time and average weekly sleep with naps, and ghrelin was significantly correlated with sleep efficiency and WASO. Figure 3A shows the mean leptin levels for half-hour increments of average nightly sleep after adjustment for age, sex, BMI, and time of storage (see Table 2). In the multiple regression model (see Table 4), there was a significant increasing trend in leptin for average nightly sleep duration (*p =* 0.01). When evaluated at the average values and sex distribution of our sample, a decrease from 8 to 5 h in average nightly sleep was associated with a predicted 15.5% decrease in leptin. Figure 3B shows the mean ghrelin levels for half-hour increments of total sleep time after adjustment for age, sex, BMI, and time of storage (see Table 2). In the multiple regression model (see Table 4), there was a significant decreasing trend in ghrelin with total sleep time (*p =* 0.008). When evaluated at the average values and sex distribution of our sample, a decrease from 8 to 5 h of polysomnographically defined total sleep time was associated with a predicted 14.9% increase in ghrelin. There was no significant correlation between sleep duration (acute or chronic) and serum adiponectin, insulin, glucose, or QUICKI. Results of our analyses were unchanged after adjusting for the weighted sampling scheme.

**Table 4:**
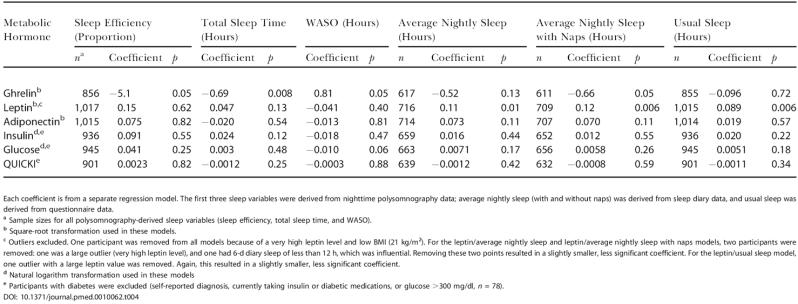
Relationships between Sleep Variables and Metabolic Hormones, Adjusted for Age, Sex, BMI, and Time of Sample Storage

Each coefficient is from a separate regression model. The first three sleep variables were derived from nighttime polysomnography data; average nightly sleep (with and without naps) was derived from sleep diary data, and usual sleep was derived from questionnaire data

^a^ Sample sizes for all polysomnography-derived sleep variables (sleep efficiency, total sleep time, and WASO)

^b^ Square-root transformation used in these models

^c^ Outliers excluded. One participant was removed from all models because of a very high leptin level and low BMI (21 kg/m^2^). For the leptin/average nightly sleep and leptin/average nightly sleep with naps models, two participants were removed: one was a large outlier (very high leptin level), and one had 6-d diary sleep of less than 12 h, which was influential. Removing these two points resulted in a slightly smaller, less significant coefficient. For the leptin/usual sleep model, one outlier with a large leptin value was removed. Again, this resulted in a slightly smaller, less significant coefficient

^d^ Natural logarithm transformation used in these models

^e^ Participants with diabetes were excluded (self-reported diagnosis, currently taking insulin or diabetic medications, or glucose >300 mg/dl, *n* = 78)

DOI: 10.1371/journal.pmed.0010062.t004

## Discussion

We found that habitual sleep duration below 7.7 h was associated with increased BMI, similar to findings in other studies including children [1,17], adolescents [5], and adults [2,3]. We also report a significant association of sleep duration with leptin and ghrelin that is independent of BMI, age, sex, SDB, and other possible confounding factors (analysis not shown for SDB and other confounders). Short sleep duration was associated with decreased leptin and increased ghrelin, changes that have also been observed in reaction to food restriction and weight loss and are typically associated with increased appetite. These hormone alterations may contribute to the BMI increase that occurs with sleep curtailment.

Previous studies have shown that both acute sleep deprivation [18] and chronic partial sleep deprivation (sleep restriction) [19] can cause a decrease in serum leptin concentrations. These studies, however, were performed under highly controlled laboratory circumstances. Our results validate the association of decreased leptin with decreased sleep time in a large sample of adults under real-life conditions and, now, indicate a role for ghrelin. Leptin deficiency increases appetite and produces obesity [8,20]. Leptin administration suppresses food intake and reduces energy expenditure [21,22]. Importantly, low leptin as observed with sleep loss has a greater impact on appetite than high leptin levels, which are associated with leptin resistance, as occurs with obesity [8].

Levels of ghrelin, a potent stimulator of appetite [23,24,25], were higher in those with shorter sleep. Ghrelin levels are also positively associated with hunger ratings [26], but decrease with increased BMI (see Table 2). In one study, after 3 mo of dietary supervision, a reduction in BMI of approximately 5% was associated with a 12% increase in ghrelin and a 15% decrease in leptin [27]. These changes, in participants of similar BMI to our sample and presumably producing increased appetite, are comparable to those observed with sleep loss of 2–3 h/night. With sleep loss, however, relatively high ghrelin and low leptin levels are associated with increased BMI. These changes can be hypothesized to play a contributory, rather than compensatory, role in the development of overweight and obesity with sleep restriction.

Our findings are strengthened by the large and well-characterized population-based sample, attention to bias and confounding factors, and in-laboratory polysomnographic data. The changes in hormones with sleep duration were consistent and of significant magnitude. They also represent the first demonstration of a correlation between peripheral hormone levels and both self-reported (questionnaire and diary data) and polysomnographically determined sleep amounts in a general population sample. While these data are more comprehensive than previous studies on this topic, some misclassification error may exist because of intra-person variability or limitations of polysomnographic measurement. Little is known about the stability of self-reported sleep duration and polysomnographic measures of sleep duration over time. We examined the stability of the self-reported sleep duration data, and found these measures to be stable. For 860 participants who completed three surveys, the mean (standard deviation) of intra-person differences in usual sleep for two 5-y periods was 0.10 (0.47) h. For 190 participants with at least three sleep diaries, the mean (standard deviation) of intra-person differences in average nightly sleep for two 4-y intervals was 0.09 (0.41) h. Furthermore, the subjectively reported hours of usual sleep and the diary-derived average nightly sleep values were highly correlated (*r =* 0.55, *p <* 0.001). One-night polysomnographically defined total sleep time had a similar intra-person mean difference (0.10 h), with a somewhat larger standard deviation (0.68) for 713 participants with at least three sleep studies.

Elevated ghrelin mainly correlated with acute sleep loss as measured by polysomnography immediately prior to blood sampling (see Table 4; Figure 3B), while reduced leptin correlated with chronic sleep restriction indicated by self-reported sleep measures (see Table 4; Figure 3A). Measures of usual and one-night polysomnographically defined sleep time were only weakly, but statistically significantly, correlated (*r =* 0.12, *p <* 0.001), supporting the concept that these measures reflect long-term and short-term changes in sleep amounts, respectively. Our findings are in agreement with the current view that leptin is important in signaling long-term nutritional status while ghrelin has a more significant role in acute hunger. The changes in leptin and ghrelin with sleep restriction could, therefore, provide a powerful dual stimulus to food intake that may culminate in obesity.

Longitudinal and intervention studies will be necessary to define further the link between sleep curtailment and increased BMI. Only total ghrelin was measured, since active octanoylated ghrelin is unstable. Although both total and active ghrelin appear to be regulated in a similar and parallel manner, future studies will need to focus on measurement of the biologically active form. Other potentially important appetite regulatory hormones, such as PYY 3–36 [28], were not measured. Measures of appetite were not included in the Wisconsin Sleep Cohort Study overnight protocol; therefore, a direct examination of the relationship between the observed hormone changes with sleep duration and alterations in appetite was not possible.

Hormone measurements were all performed on a single fasted, morning sample and may not reflect the 24-h profile. It is possible that participants with shorter sleep woke up earlier and that hormone differences may be partially related to circadian time. Leptin and ghrelin levels rise slightly during the night [29], and this could result in higher hormone levels in short sleepers. This may be an issue for ghrelin, as levels increased with acute sleep restriction. It is, however, unlikely to play a role in the leptin finding, since lower levels were found with chronic but not acute sleep restriction. Additionally, studies have shown a high correlation between morning, fasting leptin and ghrelin levels and 24-h mean profile [29,30]. We also found that the ghrelin and leptin changes were unaffected by morningness tendencies. The fact that studies investigating the diurnal profile of these hormones found similar hormonal changes over the entire 24-h period after experimental sleep restriction also corroborates our results [18,19]. The robustness of our findings and similar observations from smaller controlled studies [18,19] also suggest that our statistically significant results are unlikely to be a reflection of the number of analyses carried out.

Animal studies have suggested a link between sleep and metabolism [31,32]. In rats, prolonged, complete sleep deprivation increased both food intake and energy expenditure. The net effect was weight loss and, ultimately, death [33]. Rats fed a high protein-to-calorie diet had accelerated weight loss, compared to sleep-deprived rats fed calorie-augmented (fatty) diets [32]. Food consumption remained normal in sleep-deprived rats fed protein-rich diets, but increased 250% in rats fed calorie-rich diets. Preference for fatty foods has also been reported anecdotally in sleep-deprived humans [32,34]. Sleep deprivation may thus increase not only appetite but also preference for lipid-rich, high-calorie foods. Animal experiments that have found weight loss after prolonged sleep deprivation have to be interpreted in the context of a stressful procedure producing intense sleep debt [35,36], which may interfere with adequate food intake. From our study, we hypothesize that the moderate chronic sleep debt associated with habitual short sleep is associated with increased appetite and energy expenditure. In societies where high-calorie food is freely available and consumption uncontrolled, after milder chronic sleep restriction, the equation may be tipped towards food intake for high-calorie food rather than expenditure, culminating in obesity. Short sleepers may also have more time to overeat.

Sleep loss from a baseline of 7.7 h was associated with a dose-dependent increase in BMI. This was the predominant effect in a population increasingly curtailing sleep [37]. Sleep greater than 7.7 h, however, was also associated with increased BMI. Patients with SDB (a pathology associated with increased BMI) may spend a longer time in bed to compensate for fragmented sleep; however, controlling for AHI did not change the curvilinear BMI–sleep association. Another possibility is that in long sleepers, reduced energy expenditure due to increased time in bed has a greater impact than reduced food intake. In favor of this hypothesis, long sleepers exercise less [38]. In our data, we found that the odds ratio of high levels of self-reported exercise (>7 h/wk), based on a single survey question, decreased with increased sleep time, but controlling for this variable also did not change our findings (analyses not shown).

Insulin resistance with sleep deprivation has been reported in a laboratory study of young, healthy volunteers [39]. When controlling for BMI, we found no significant correlation between insulin, glucose, or adiponectin levels and various measures of sleep duration. Also, there was no significant correlation between QUICKI (or the homeostatic model assessment HOMA [16]; data not shown) and sleep duration. This may be due to difficulties in detecting small effects on glucose tolerance under less-controlled conditions of large population studies.

Our results demonstrate an important relationship between sleep and metabolic hormones. The direct effect of chronic partial sleep loss on food intake, energy expenditure, and obesity now needs to be explored. Altering sleep duration may prove to be an important adjunct to preventing and treating obesity.

**Patient Summary**

**Why Was This Study Done?**

Recent studies have shown that there is a link between sleeping less and gaining weight. It isn't clear why there is this link—perhaps, for example, those who are awake in the middle of the night tend to head for the refrigerator for a snack. Another possibility is that the amount of sleep that we have might affect the hormones that control our appetite. We know that extreme sleep deprivation affects the level of leptin, a hormone that controls appetite. Here, the researchers wanted to study levels of leptin and other appetite hormones under more normal conditions, in people with a range of sleeping habits.

**What Did the Researchers Do?**

The researchers studied participants in a large sleep study that has been going on in Wisconsin for over 15 years. These participants have been filling out questionnaires about their sleep habits and their health in general, have kept sleep diaries, and have occasionally spent a night in the laboratory, where researchers studied their sleep in more detail. After sleeping overnight in the laboratory, the participants gave blood samples, which were tested for hormones.

**What Did They Find?**

The researchers found that people who slept less were on average heavier. And people who slept less had lower levels of leptin and higher levels of ghrelin, another hormone that controls food intake.

**What Does This Mean?**

The combination of low leptin and high ghrelin is likely to increase appetite. In other words, short sleep might stimulate appetite, which increases weight.

**What Next?**

Future studies need to examine the effect of regular short sleeping hours on appetite, food intake, and obesity. These studies could help to answer the question of whether the rise in obesity in many societies is partly due to the fact that people are sleeping less. And it seems well worth testing whether increasing sleep to seven or eight hours per night could help people to lose weight.

**Additional Online Information**

National Sleep Foundation Web page on obesity and sleep: http://www.sleepfoundation.org/features/obesity.cfm

World Federation of Sleep Research Societies: http://www.wfsrs.org/homepage.html

Red en Medicina del Sueño: http://www.rems.com.ar/

European Sleep Research Society: http://www.esrs.org/

## Abbreviations

AHI: apnea-hypopnea index
BMI: body mass index
BUN: blood urea nitrogen
HDL: high-density lipoprotein
QUICKI: quantitative insulin sensitivity check index
SDB: sleep-disordered breathing
WASO: wake after sleep onset
